# Elevation of Matrix Metalloproteinases in Different Areas of Ascending Aortic Aneurysms in Patients with Bicuspid and Tricuspid Aortic Valves

**DOI:** 10.1100/2012/806261

**Published:** 2012-05-03

**Authors:** Salah A. Mohamed, Frank Noack, Kerstin Schoellermann, Anje Karluss, Arlo Radtke, Detlev Schult-Badusche, Peter W. Radke, Bjoern E. Wenzel, Hans H. Sievers

**Affiliations:** ^1^Department of Cardiac and Thoracic Vascular Surgery, University Clinic of Schleswig-Holstein Campus Luebeck, Ratzeburger Allee 160, 23538 Luebeck, Germany; ^2^Institute of Pathology, UKSH, Campus Luebeck, Ratzeburger Allee 160, 23538 Luebeck, Germany; ^3^Department of Medical Medicine II, UKSH, Campus Luebeck, Ratzeburger Allee 160, 23538 Luebeck, Germany; ^4^MKI, UKSH, Campus Luebeck, Ratzeburger Allee 160, 23538 Luebeck, Germany

## Abstract

Our aim is to investigate the elevation of matrix proteins in tissues obtained from distal, above the sinotubular junction (proximal), concave, and convex sites of aneurysms in the ascending aorta using a simultaneous multiplex protein detection system. Tissues were collected from 41 patients with ascending aortic aneurysms. A total of 31 patients had a bicuspid aortic valve (BAV), whereas 10 had a tricuspid aortic valve (TAV). Concave and convex aortic site samples were collected from all patients, whereas proximal and distal convexity samples were obtained from 19 patients with BAV and 7 patients with TAV. Simultaneous detection of matrix metalloproteinases (MMPs) and their inhibitors (TIMPs) was performed at each of the four aortic sites. MMP-2 levels were higher in the concave aortic sites than in the convex aortic sites. In contrast, MMP-8 levels were higher in the convex sites than in the concave sites, as were MMP-9 levels. In both BAV and TAV patients, TIMP-3 levels were higher in the concave sites than in the convex sites. However, TIMP-2 and TIMP-4 levels were significantly elevated in the sinotubular proximal aorta of BAV patients. Simultaneous detection of MMPs and TIMPs revealed different levels at different aortic sites in the same patient.

## 1. Introduction

Aortic aneurysms are characterized by weakened and distorted arterial architecture and are relatively common causes of death because of arterial dissection or rupture. These aneurysms are common among the elderly. A bicuspid aortic valve (BAV), the most common congenital cardiac malformation, is associated with ascending thoracic aneurysms and appears to reflect a common developmental defect [1, 2]. Both cellular and extracellular processes are involved in the pathogenesis of ascending thoracic aortic aneurysms in patients with BAV [3–6]. On an average, patients with BAV undergo surgery to correct abnormal valve morphology and/or to treat complications associated with the diseased aortic valve a decade earlier than patients with a normal tricuspid aortic valve. Replacing the aortic valve in patients with BAV does not prevent progressive dilation of the aortic root and ascending aorta [7].

Many studies have demonstrated abnormalities in matrix metalloproteinases (MMPs) and tissue inhibitors of metalloproteinases (TIMPs) in aneurysmal tissues [8–10]. Using tissue microarray techniques, Koullias et al. [10] detected significantly higher levels of MMP-2 and MMP-9 in BAVs than in TAVs; in this study, MMP-2, MMP-9, and TIMP-1 levels were significantly higher in BAV tissues than in all other tissues (control and TAV tissues). Lemaire et al. [11] found a lack of inflammatory activity, an increased level of MMP-2, and normal expression of MMP-9, TIMP-1, and TIMP-2 in aneurysmal tissues obtained from patients with BAV. In contrast, in aneurysmal tissues obtained from patients with TAV, they found increased inflammatory activity and MMP-9 levels [11]. They also demonstrated an increased incidence of cultured vascular smooth muscle cell (VSMC) loss among individuals with BAV and Marfan's syndrome (MFS) when compared with that in healthy controls and suggested a possible link between MMP-2 upregulation and VSMC apoptosis in MFS. There are certainly similarities in the histology of aneurysmal aorta tissues in MFS and BAV patients [8]. In MFS, a mutation in the gene for the extracellular matrix protein, fibrillin-1, leads to dysregulation of transforming growth factor beta signaling [12]. Although many studies have demonstrated MMP elevation in aneurysms, matrix protein expression appears to vary between aneurysmal tissues [13].

This study aimed to identify the matrix proteins present at different sites in aortic aneurysms. A system for simultaneously detecting six MMPs and their four inhibitors at different sites in the thoracic aorta was developed. This method is as accurate as older methods, the advantage being that it minimizes the errors associated with those methods.

## 2. Materials and Methods

### 2.1. Study Protocol

**Figure 1:**
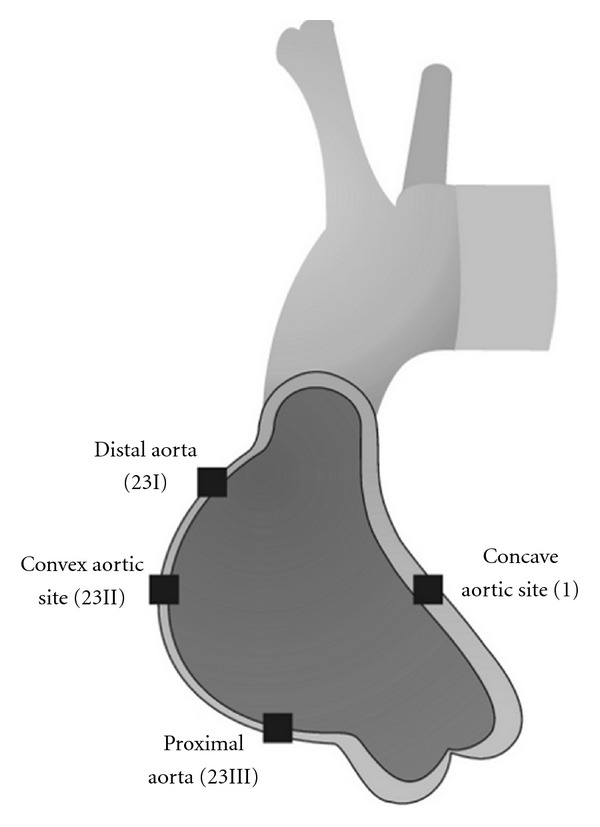
Schematic representation of ascending aneurysms. Resected tissue from the distal, convex, proximal, and concave aortic sites used for the analysis. Overall detection of MMPs and TIMPs at the concave and convex aortic sites.

The study protocol was approved by the Institutional Ethics Committee, and written informed consent of each patient was obtained. In cases of ascending aorta replacement surgery, 3 × 3 mm distal specimens were obtained for protein multiplex analysis from four different sites of ascending aortic aneurysms: concave, convex, distal, and proximal aortic sites (Figure 1). The 3 × 3 mm specimens were then washed in wash buffer, transferred to Bioplex cell lysis buffer (BioRad Laboratories, Hercules, CA, USA), and frozen in liquid nitrogen. Specimens were stored at −80°C until further use. Patient demographics are shown in Table 1.

### 2.2. Tissue Preparation for the Multiplex Method

Tissue samples were thawed in 500 *μ*L lysis solution and ground with an Ultra-turrax T8 homogenizer (IKA Labortechnik, Staufen, Germany). After about 20 strokes, the debris and lysates were separated by centrifugation at 4500 ×g for 5 min. The supernatant was immediately processed to measure the amount of protein, MMPs, and TIMPs. The samples were kept on ice during all procedures to avoid protein denaturation.

The multiplex method used color-coded bead sets conjugated with specific reactants, each of which was specific for a different target molecule. The principle is that of a capture sandwich (immuno)-assay, but 96-well microtiter plates with filter bottoms are used. Antibodies specifically directed against the analytes (MMPs and TIMPs) of interest were covalently bound to color-coded 5.6 *μ*m polystyrene beads and mixed with a defined sample volume containing equal amounts of total protein but an unknown amount of MMPs and TIMPs. A standard curve was obtained for each run by diluting of a standard cocktail designed for different panels of targets provided by the manufacturer (BioRad Laboratories, Hercules, CA, USA). After three wash cycles to remove unbound protein (via a vacuum manifold through the plate's filter bottom), biotinylated detection antibodies specific for a second epitope of the analytes were added. The mixtures were then washed another three times and streptavidin phycoerythrin (S-PE) added for specific binding to biotin. The mixture in each well was then drawn up into a dual-laser, flow-based luminometer using Luminex technology (Bioplex, BioRad Laboratories, Hercules, CA, USA). Briefly, one laser identified the color-coded beads and sorted the targets while the other laser determined the magnitude of the S-PE value, which was directly proportional to the amount of analyte (MMP or TIMP). MMP-1, MMP-2, MMP-8, MMP-9, MMP-12, and MMP-13 as well as TIMP-1, TIMP-2, TIMP-3, and TIMP-4 were detected using a Human MMP Fluorokine MultiAnalyte Profiling (FMAP) Base Kit and other appropriate kits for this panel of targets from R&D Systems (Minneapolis, MN, USA); all were applied in accordance with the manufacturer's instructions.

### 2.3. Statistics

All data examined are expressed as mean ± standard error mean or standard deviation. Statistical analysis of the data was performed using Excel/WinSTAT software (R. Fitch software, Bad Krozingen, Germany). Differences among groups were assessed with Wilcoxon tests, followed by a Mann-Whitney *U* test. Significant differences were defined by *P* < 0.05.

## 3. Results

### 3.1. Study Subjects

A total of 41 patients were included in the analysis; concave and convex aortic site samples were collected from all patients, whereas proximal and distal convexity samples were obtained from 19 patients with BAV and 7 patients with TAV. The BAV group of 31 patients comprised 24 males (77%) and 7 females, while 7 of the 10 TAV patients were male (70%). The age distribution was considerably different between the two groups. BAV patients had a mean age of 50.9 ± 12.9 years and were significantly younger than the TAV patients, whose mean age was 63.2 ± 8.2 years (*P* = 0.006). The mean aortic diameter was, however, comparable between the groups (52.7 ± 4.9 mm versus 56.7 ± 6.6 mm). There were also differences in the type of aortic valve disease identified between the two groups. While the BAV group included 10 patients with aortic valve insufficiency (32%), 3 patients with stenosis (10%), and 18 patients with a combination of both (58%), all TAV patients suffered from aortic valve insufficiency.

### 3.2. Simultaneous Detection of MMPs and TIMPs Using Multiplex System Analyses

Multiplex system (Bio-Plex) analyses were used to determine protein concentrations of MMP-1, MMP-2, MMP-8, MMP-9, MMP-12, and MMP-13 as well as concentrations of TIMP-1, TIMP-2, TIMP-3, and TIMP-4 at different sites of an aneurysmatic aorta ascendens (Figure 1). Results were analyzed in both patient groups according to the following classifications:

- areas 1 (the concave aortic site) and 23II (the convex aortic site),
- areas 1 and 23I (the distal aorta) and 23III (the proximal aorta),
- areas 23I and 23II, 23II and 23III, and 23I and 23III,
- comparison between every site in both BAV and TAV patients.

Analyses were carried out simultaneously for all MMPs and TIMPs. Subsequently, the patients were divided into different groups on the basis of age, diameter of aneurysm, sex, and aortic valve disease. The above categories (a), (b), (c), and (d) were used to compare these groups. The groups were also compared with each other (e).

MMP-1, MMP-12, and MMP-13 were undetectable in most cases because of very low concentrations. Therefore, they were omitted from the final calculations.

Comparison (41 patients) between areas 1 and 23II revealed significantly higher levels of MMP-8 and MMP-9 in area 23II, a dilated aortic site, than in area 1 in all patients (*P* = 0.001, *P* = 0.007). MMP-2 and TIMP-3 levels, however, were higher in area 1 (*P* = 0.04, *P* = 0.0007; Table 2).

### 3.3. Detection of MMPs and TIMPs in BAV versus TAV Patients (Concave, Distal, and Proximal Aortic Sites)

For patients with BAV, the aneurysmal areas 23I and 23III had significantly higher MMP-8 and MMP-9 concentrations (MMP-8: *P* = 0.003, *P* = 0.02; MMP-9: *P* = 0.01, *P* = 0.04) than those in area 1, whereas TIMP-3 levels were higher in area 1 (*P* = 0.004 versus area 23I, *P* = 0.03 versus area 23III).

TAV patients had higher TIMP-3 levels in nondilated area 1 than in the dilated area 23II (*P* = 0.008). When areas 23I, 23II, and 23III were compared, the BAV group was found to have significantly higher TIMP-3 levels than those in the TAV group (*P* = 0.02).

### 3.4. Cross-comparisons between the BAV and TAV Groups

#### 3.4.1. Elevation of MMPs and TIMPs Based on the Diameter of the Aortic Aneurysm

To classify patients on the basis of the aortic aneurysm diameter, a threshold diameter between 54 mm and 55 mm was chosen after considering that the mean diameter was 53.7 ± 5.6 mm. Most TAV-associated aneurysms had larger diameters than the BAV-associated aneurysms. Therefore, all TAV patients were in the group with aneurysms ≥55 mm.

**Figure 2:**
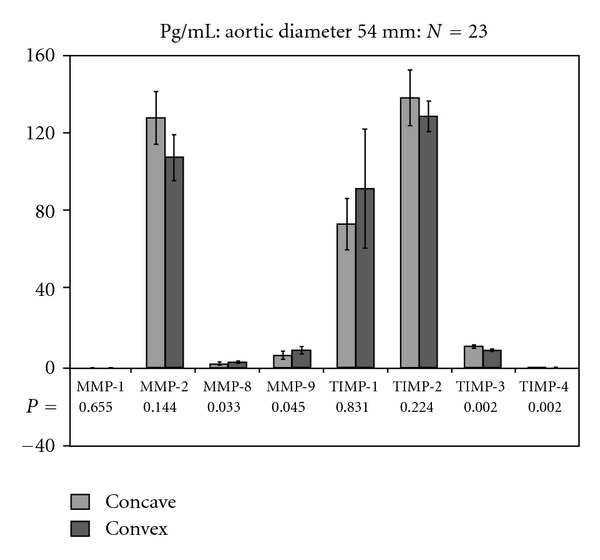
Detection of MMPs and TIMPs in BAV and TAV patients with aneurysms ≤54 mm in diameter.

When area 1 and area 23II were compared in all patients with an aneurysm of ≤54 mm diameter (*n* = 23), higher MMP-8 and MMP-9 levels were observed in area 23II than in area 1. On the other hand, expressions of TIMP-3 and TIMP-4 were significantly lower in area 23II than in area 1 (*P* = 0.002; *P* = 0.002; Figure 2).

**Figure 3:**
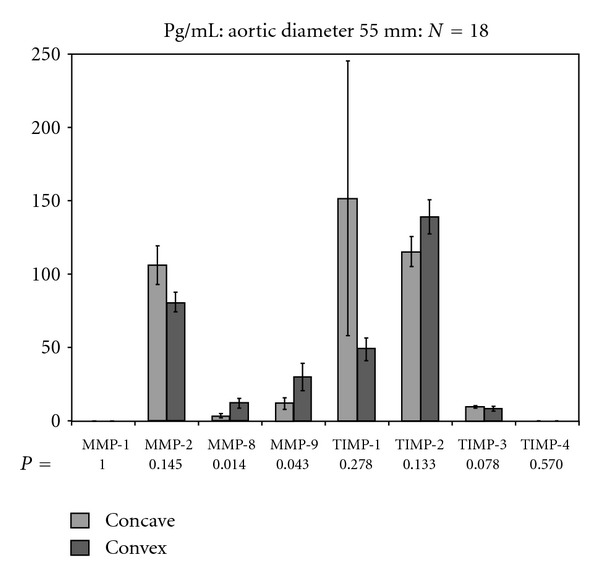
Detection of MMPs and TIMPs in BAV and TAV patients with aneurysms ≥55 mm in diameter.

Patients with an aneurysm ≥55 mm in diameter (*n* = 18) had considerably higher MMP-8 and MMP-9 levels in aneurysmal area 23II than in area 1 (*P* = 0.01; *P* = 0.04) (Figure 3).

#### 3.4.2. Elevation of MMPs and TIMPs with Advancing Age

Patients were classified into two groups according to their age. Due to the significantly higher age of the TAV patients (63.2 ± 8.2 years), the mean age of the BAV patients (50.9 ± 12.9 years, *P* = 0.006) was used as a reference, with 51 years considered the age threshold. All TAV patients were sorted into the group aged older than 51 years (Table 3).

Within the BAV group, the patients aged ≤51 years had significantly lower expression of TIMP-3 in the dilated area 23II than in area 1 (10.35 ± 3.4 pg/mL, *P* = 0.01).

Results for the TAV group and the comparison between patients of different ages were omitted because of their older age.

In BAV patients aged ≥52 years, significantly elevated MMP-8 and MMP-9 levels were detected in area 23II when compared with those in area 1 (10.84 ± 13.92 pg/mL, *P* = 0.002; 25.45 ± 37.84, *P* = 0.01; Table 4).

When BAV and TAV patients were compared, elevated TIMP-3 levels were observed in area 23II in BAV patients (*P* = 0.02).

#### 3.4.3. Elevation of MMPs and TIMPs Based on Sex

No female TAV group was established because of the low number of female patients. Analyses of the male BAV group revealed significantly increased MMP-8 and MMP-9 levels in dilated area 23II than in area 1 (*P* = 0.005, *P* = 0.01), whereas TIMP-3 concentrations were higher in area 1 (*P* = 0.03).

Comparisons between the two male groups did not reveal any significant results.

Because of the small number of female BAV patients, only areas 1 and 23II could be compared. These were not significantly different.

Comparison of male and female BAV patients revealed significantly higher TIMP-1 levels in area 23II of female patients (*P* = 0.03).

#### 3.4.4. Elevation of MMPs and TIMPs Based on Aortic Valve Disease

Classification of patients according to the type of aortic valve disease was restricted by the low number of patients with isolated stenosis. Therefore, only BAV patients with aortic valve insufficiency or a combination of insufficiency and stenosis were considered.

All TAV patients were included in the insufficiency group.

The BAV group with aortic valve insufficiency had increased TIMP-3 levels in area 23II than in area 1 (*P* = 0.02). No further comparisons were made because of small sample number.

BAV patients with aortic valve insufficiency had increased TIMP-3 levels in aortic area 23II when compared with those in TAV patients with aortic valve insufficiency (*P* = 0.03).

The BAV group with a combination of insufficiency and stenosis had significantly higher MMP-8 and MMP-9 levels in aneurysmal area 23II than in area 1 (*P* = 0.004, *P* = 0.007).

## 4. Discussion

In the literature, reports of the matrix protein expression in aneurysmal tissues have documented varied results [8, 10, 11, and 13]. In accordance with the published data, we observed the profiles of six MMPs and their four inhibitors using a simultaneous detection system at four different aortic sites (concave, convex, distal, and proximal sites) of ascending aortic aneurysms. MMP-2, MMP-8, MMP-9, TIMP-1, TIMP-2, TIMP-3, and TIMP-4 were all detected and quantified. However, protein concentrations of MMP-12 and MMP-13 were very low and therefore omitted. These areas were combined in 41 patients (31 BAV and 10 TAV). The patients were divided into groups on the basis of age, ascending aneurysm diameter, sex, and valve malformation. When the entire patient dataset was assessed, significantly higher levels of MMP-8 and MMP-9 were detected in the dilated aortic areas (convexity) and in the distal and proximal aorta than in the concave aortic sites in all patients. In contrast, MMP-2 and TIMP-3 levels were elevated in the concave sites.

Comparison of the distal, convex, and proximal aortic sites revealed no significant differences among BAV patients. However, among TAV patients, TIMP-3 levels were higher in the concave sites than in the convex, distal, and proximal aortic sites. Comparisons between the proximal and distal aortic sites revealed an elevated TIMP-4 concentration in the distal aorta of TAV patients. Significantly higher TIMP-3 levels were revealed in four aortic sites of BAV patients (*n* = 19) than in those of TAV patients (*n* = 7). Furthermore, MMP-2, TIMP-2, TIMP-3, and TIMP-4 concentrations were also much higher in the proximal aorta of BAV patients than in that of TAV patients.

Patients were categorized into two groups on the basis of their age. Because of the significantly older age of TAV patients, 51 years was considered the age threshold. Consequently, all TAV patients were placed in the group aged >51 years.

Within the BAV group, patients aged ≤51 years had significantly lower TIMP-3 expression in the distal and convex aortic sites than in the concave site. Comparison between the aneurysmal areas (distal, convex, and proximal aortic sites) revealed increased levels of MMP-8 and TIMP-4 in the distal aorta than those in the proximal aorta. Significantly elevated MMP-8 and MMP-9 levels were also detected among BAV patients aged ≥52 years in the concavity compared with younger patients.

To classify patients on the basis of the aortic aneurysm diameter, a threshold between 54 and 55 mm was chosen; all TAV patients were allocated to the group with aneurysms ≥55 mm.

Higher MMP-8 and MMP-9 levels were detected in the convex aortic sites than in the concave aortic sites of BAV patients with aneurysms ≤54 mm in diameter. In contrast, TIMP-3 and TIMP-4 levels were significantly lower in the convex aortic sites.

MMP-8 and MMP-9 levels were also elevated in the distal aorta compared with those in the concave aortic sites, but TIMP-3 levels were elevated in the concave sites when compared with the distal and proximal aortic sites.

Patients with BAV and aneurysms ≥55 mm in diameter had considerably higher levels of MMP-8 and MMP-9 in the convex sites than in the concave sites.

Elevated MMP-8 levels were detected in the proximal and distal aortic sites than in the concave sites; TIMP-3 levels were higher in the proximal aorta than in the distal aorta.

The TAV group had higher TIMP-3 expression in the concave sites than in the convex sites.

Analysis of the male BAV group revealed significantly increased MMP-8 and MMP-9 levels in the dilated convex sites than in the concave sites, which had elevated TIMP-3 levels. Among female BAV patients, the distal and the proximal aortic sites had elevated MMP-8 and MMP-9 levels compared with those in the concave sites, whereas TIMP-3 levels were lower in the former sites. Comparison between the male BAV groups revealed elevated TIMP-3 levels in the convex sites than in the distal aortic sites. Among the male TAV group, comparisons between the distal, convex, and proximal sites did not reveal any significant differences.

The BAV group with aortic valve insufficiency had elevated TIMP-3 levels in the convex aortic sites than in the concave aortic sites. In contrast to TAV patients with aortic valve insufficiency, BAV patients with insufficiency had increased TIMP-3 concentrations in the convex aortic sites.

The BAV group with a combination of aortic valve diseases had significantly increased MMP-8 and MMP-9 levels in the convex aortic sites. TIMP-3 levels, on the other hand, were higher in the convex aortic sites than in the distal aorta. Of note, some studies did not observe differences in gene expression of MMPs and TIMPs between patients with BAV and TAV [13, 14]. Therefore, we listed the limitations of our study as follows: we were unable to collect appropriate control samples, especially those samples of nonaneurysmal and with excluding atherosclerotic aortas. In addition, we did not detect significant correlation between the histopathological features and elevation of MMPs and TIMPs (data not shown). However, we believe that this study clearly reflected the difference in the elevation of MMPs and TIMPs protein levels in tissue of thoracic ascending aneurysms of patients with BAV and TAV.

## 5. Conclusions

Although we did not measure these proteins in blood or body liquids, the obtained results indicate that MMPs and matrix proteins can vary in concentration in ascending aortic aneurysms in patients with BAV. Many studies support a genetic origin of BAV [15–19]; however, hemodynamics can also play a role in the pathogenesis of BAV and/or ascending aortic aneurysms. Recent studies have indicated that aortic wall shear stress differs locally between BAV and control patients when examined by magnetic resonance imaging [20, 21]. Therefore, we believe that simultaneous detection of MMPs and TIMPs offers a powerful tool for systematic investigations to further our understanding of the complex pathogenesis of thoracic aortic aneurysms.

## Figures and Tables

**Table 1:** Patient demographics and clinical characteristics.

	*N*	Age (*Y*)	Sex (M/F)	A. Ømm	AVS	AVI	AV com.
All	41	53.9 ± 13.1	31/10	53.7 ± 5.6	3	20	18
BAV	31	50.9 ± 13.1	24/7	52.7 ± 4.9	3	10	18
TAV	10	63.2 ± 8.2	7/3	56.7 ± 6.6	0	10	0

Absolute values (±SD): A. Ømm: ascending aorta in mm: AVS: aortic valve stenosis: AVI: aortic valve insufficiency: AV com.: aortic valve combined.

**Table 2:** Overall detection of MMPs and TIMPs using the multiplex system.

*N* = 41			*N* = 41			*P*
Concave	Average (pg/mL)	SD±	Convex	Average (pg/mL)	SD±	
MMP-1	0.004	0.018	MMP-1	0.005	0.028	n.s.
MMP-2	118.536	61.232	MMP-2	96.035	47.555	0.04
MMP-8	2.760	5.147	MMP-8	7.056	10.626	0.001
MMP-9	8.923	13.249	MMP-9	18.279	28.544	0.007
TIMP-1	107.840	265.137	TIMP-1	73.168	112.832	n.s.
TIMP-2	128.211	59.117	TIMP-2	133.354	42.847	n.s.
TIMP-3	10.417	3.413	TIMP-3	8.810	5.028	0.0007
TIMP-4	0.216	0.084	TIMP-4	0.207	0.091	n.s.

n.s.:not significant.

**Table 3:** Age differences and detection of MMPs and TIMPs in BAV and TAV patients aged ≤51 years.

Average pg/mL	BAV (*n* = 16)			TAV (*n* = 0)		
	Concave	Convex	*P*	Concave	Convex	*P*
MMP-1	0.006 ± 0.02	0.01 ± 0.04	n.s.	—	—	—
MMP-2	109.8 ± 59.5	100.3 ± 56.6	n.s.	—	—	—
MMP-8	2.4 ± 4.7	2.3 ± 2.2	n.s.	—	—	—
MMP-9	7.4 ± 10.7	8.7 ± 9.2	n.s.	—	—	—
TIMP-1	52.8 ± 37.8	58.5 ± 64.9	n.s.	—	—	—
TIMP-2	135.0 ± 71.5	125.2 ± 42.3	n.s.	—	—	—
TIMP-3	10.3 ± 3.4	8.2 ± 3.2	0.01	—	—	—
TIMP-4	0.2 ± 0.08	0.2 ± 0.07	n.s.	—	—	—

n.s.: not significant.

**Table 4:** Age differences and detection of MMPs and TIMPs in BAV and TAV patients aged ≥52 years.

Average (pg/mL)	BAV (*n* = 15)			TAV (*n* = 10)		
	Concave	Convex	*P*	Concave	Convex	*P*
MMP-1	0.001 ± 0.005	0.000	n.s.	0.005 ± 0.01	0.003 ± 0.006	n.s.
MMP-2	136.2 ± 61.1	104.7 ± 43.8	n.s.	105.7 ± 64.0	76.1 ± 33.1	n.s.
MMP-8	1.7 ± 1.9	10.8 ± 13.9	0.002	4.8 ± 8.2	8.9 ± 11.2	n.s.
MMP-9	6.7 ± 8.1	25.4 ± 37.8	0.01	14.6 ± 20.8	22.7 ± 31.3	n.s.
TIMP-1	201.8 ± 428.7	61.4 ± 30.1	n.s.	54.8 ± 30.7	114.2 ± 213.7	n.s.
TIMP-2	132.3 ± 50.8	141.9 ± 34.2	n.s.	111.1 ± 50.5	133.5 ± 55.6	n.s.
TIMP-3	11.2 ± 3.4	11.0 ± 7.1	n.s.	9.2 ± 3.3	6.4 ± 1.0	0.02
TIMP-4	0.24 ± 0.07	0.24 ± 0.11	n.s.	0.18 ± 0.10	0.16 ± 0.05	n.s.

n.s.: not significant.
